# External Reorganization Energy upon Charge Transfer Reactions in Mildly Polar Media: The Case of Naphthalene in Tetrahydrofuran

**DOI:** 10.1021/acs.jpclett.5c01328

**Published:** 2025-06-24

**Authors:** Francesco Ambrosio, Alessandro Landi, Michele Loriso, Anna Leo, Andrea Peluso

**Affiliations:** † Dipartimento di Scienze di Base e Applicate (DISBA), Università degli Studi della Basilicata, Viale dell’Ateneo Lucano, 10-85100 Potenza, Italy; ‡ Dipartimento di Chimica e Biologia Adolfo Zambelli, Università di Salerno, Via Giovanni Paolo II, I-84084 Fisciano (SA), Italy

## Abstract

External reorganization energy, λ_ext_, is of paramount importance in condensed-phase electron transfer (ET) processes, but its precise determination remains a challenge. We here combine classical molecular dynamics with advanced electronic-structure calculations and the thermodynamic integration technique to calculate λ_ext_ for a mildly polar solvent, tetrahydrofuran (THF), in ET reactions involving the (NAP/NAP^–^) redox couple (NAP = naphthalene), a system widely studied in this context. First, we simulate the structural and electronic properties of liquid THF, as well as those of NAP and NAP^–^ solutions, in excellent agreement with available measurements. Then, from the calculated vertical and adiabatic energy levels, we determine the values of λ_ext_ associated with the reduction of NAP and the oxidation of NAP^–^. We observe a clear asymmetry in the solvent response for the two processes, which could not be captured by either the Marcus approximation or using standard implicit solvent models. Finally, we identify the different contributions to λ_ext_ that are at the root of nonlinear solvent response, including dipole-charge interactions and effects arising from induced polarization. These interactions are found to be most significant in the first solvation shell, particularly for a limited number of solvent molecules closest to the solute.

## Graphical abstract

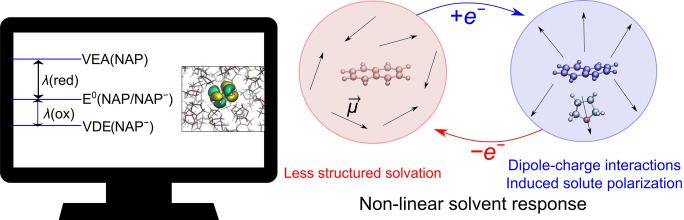

Solute–solvent interactions play a central role in electron transfer (ET) reactions in the condensed phase.^1^ The seminal work of ET in polar media developed by Marcus^2,3^ theorizes that the electron is transferred from the donor to the acceptor particle through thermal fluctuations of the medium, which modify the energy difference between their electronic states. Within this physical picture, the polarization of the medium surrounding the solute, which, in principle, derives from the translational, rotational, and vibrational degrees of freedom of solvent molecules, is encoded into a single reaction coordinate. This accounts for the so-called external or solvent reorganization energy (λ_ext_)^1–3^ and can be defined as the solvent free energy contribution to bring the reactants from their equilibrium configuration to those of the products. Extensions of Marcus theory^4,5^ accounting for quantum effects still underline how λ_ext_ influences ET, highlighting its role in modulating ET rates.^6,7^

Therefore, the accurate modeling of λ_ext_ is particularly critical for understanding ET in condensed phases: Ignoring or poorly approximating this quantity can lead to significant discrepancies between theory and experiment^8,9^ and to misinterpretation of the role of the medium in the ET process and of the temperature-dependence of the reaction.^6,7^ Unfortunately, the direct experimental determination of λ_ext_ is not straightforward and the literature is sparse: a variety of methods has been deployed to estimate this quantity from measurements, including (i) analysis of optical charge transfer bands in organic molecules,^10^ (ii) photoelectron spectra and electron attachment measurements,^8^ (iii) evaluation of the dynamic Stoke shift,^11^ and (iv) fitting emission spectra with a semiclassical Marcus equation.^12,13^ In this framework, while the solute/internal reorganization energy can be easily and accurately evaluated by standard quantum mechanics (QM) calculations, a reliable computational estimate of λ_ext_ is far from obvious.

Various methods have been developed to tackle this problem, mainly based on an implicit description of the solvent. The widely popular polarizable continuum model (PCM)^14,15^ as well as Marcus nonequilibrium approach,^2,3^ treat the solvent as a continuum dielectric with a cavity wherein the solute molecule is placed.^2,3,14–18^ These continuum models usually provide reasonable results for highly polar solvents and ionic liquids, qualitatively in line with those achieved from classical molecular dynamics (MD) simulations with an explicit treatment of solvent molecules.^19–24^ In this regard, we note that *Ab initio* MD simulations on periodic supercells have been deployed to calculate reorganization energies mainly in aqueous solution. However, the lack of proper treatment of electrostatic finite size effects and the use of approximate density functionals allowed only for a qualitative analysis, thus impeding a direct comparison with the performance of implicit methods.^25,26^

Nevertheless, models of solvation based on a dielectric continuum, which share the same spirit of the original Marcus approach, overlook the molecular nature of solute–solvent interactions, entailing local rearrangements of solvent molecules around the solute, the so-called microsolvation. Actually, this may represent the most important solvent effect in a wide range of ET reactions.^27–33^ Jen and Warshel have also shown that both strongly and weakly coupled vibronic modes are needed to correctly define the solvent bath.^34^ This is consistent with the results of MD simulations showing that (i) rearrangements in the first and second solvent shells provide the main contributions to λ_ext_ and (ii) molecular motions of a few solvent molecules (even one or two) closest to the solute can well represent the whole nuclear changes occurring upon variation of the solute charge state..^29–31^

Moreover, the accuracy of implicit models in determining λ_ext_ for less polar environments has been questioned.^35,36^ Holroyd and Miller estimated solvent reorganization energies in nonpolar liquids as large as 0.4 eV by fitting measured rates of electron attachment to molecules, a result in glaring disagreement with the predictions of implicit models of solvation.^9^ Very recently, Hsu et al.^8^ highlighted that implicit methods often severely underestimate λ_ext_ in apolar organic crystals, because molecular-level interactions and dynamic contributions are ignored. Their MD simulations revealed substantial deviations from continuum predictions and early MD-based estimates,^37^ suggesting the need for explicit modeling or hybrid approaches. Some of us also have shown that classical MD simulations need to be complemented with *ab initio* electronic-structure calculations to study the environment response in acene crystals and estimated external reorganization energies up to 80 meV, notwithstanding the apolar nature of such systems.^38,39^ The energy contribution was related to local rearrangements of the first neighbors surrounding a molecular polaron, in a fashion similar to the electrostriction mechanism proposed by Matyushov.^36,40,41^

Herein, we evaluate the solvent reorganization energy of liquid tetrahydrofuran ( l -THF) upon variation of the charge state of a solute (naphthalene). To this end, we employ a novel computational protocol based on molecular dynamics (MD) with quantum mechanically derived force fields (QMD-FF)^42–44^ which allows for an accurate, explicit treatment of solvent molecules, and *ab initio* electronic-structure calculations at the Koopman’s compliant density functional theory (KC-DFT) level of theory for the correct evaluation of the energetics and of the electronic levels.^45–47^ In particular, we choose THF as a solvent, because it is representative of a weakly polar environment, an intermediate regime whose properties have been found to differ from both highly polar and apolar solvents;^48–50^ for this reason, it stands as an interesting model system to test the accuracy of different computational methodologies in determining the solvent reorganization energy and the structural rearrangements associated with it. l -THF has also been widely used as a solvent in studies on ET reactions.^30,51–53^ As a solute, we choose naphthalene (NAP) and study the reorganization of the solvent related to its reduction to NAP^–^ and with the inverse process, i.e. oxidation of the radical anion. The NAP/NAP^–^ redox couple has been widely studied in the context of intramolecular ET reactions in THF^6,7,9,51^ and, very recently, the electronic properties of the NAP/ l -THF solutions have been thoroughly characterized via liquid jet photoemission spectroscopy,^54^ thus providing us with a reliable experimental benchmark for our methodology. Furthermore, since NAP does not include any polar functional group, it represents an ideal system also to clearly disentangle the different contributions to solvent reorganization upon reduction/oxidation.

We first describe the structural and electronic properties of l -THF. To this end, we carry out a classical MD simulation with a QM-derived force field^42,55,56^ on an atomistic model of l -THF, using the gromacs 2020.5 software.^57^ In particular, we consider a cubic supercell with side *a* = 20 Å containing 60 THF molecules (780 atoms), corresponding to the experimental density at room temperature of 0.88 g/cm^3^, cf. Figure 1 (a) and Supporting Information (SI, section S1) for details of the simulation. In Figure 1 (b), we present the oxygen–oxygen radial distribution function (RDF), *g*_OO_(*r*), achieved from our MD simulation and compare it with the experiment^58^ and previous computational results.^59^ The most characteristic feature of *g*_OO_(*r*) is the marked peak at 4.6 Å, which is correctly reproduced by our model while the largest statistically relevant difference is found in the initial rise of the RDF (see SI, Figure S2). This ensures that our computational protocol fairly describes the structural properties of l -THF. We remark that, as discussed in a previous study,^59^ the noticeable difference between computed and experimental curves might be ascribed to error accumulation in the procedure employed to derive the RDF from partial structure factors, which in turn are extracted from neutron diffraction data using inverse methods. Besides, the separation of intramolecular and intermolecular contributions in the experimental data is complex and prone to errors. In this regard, simulations suggest that intramolecular effects might be overestimated in experimental RDFs, leading to artificial peaks at short distances.^59^ In light of this discussion, our agreement with accurate ab initio calculations [green and red RDF curves in Figure 1 (b)][Bibr ref59] ensures the reliability of our approach.

**Figure 1:**
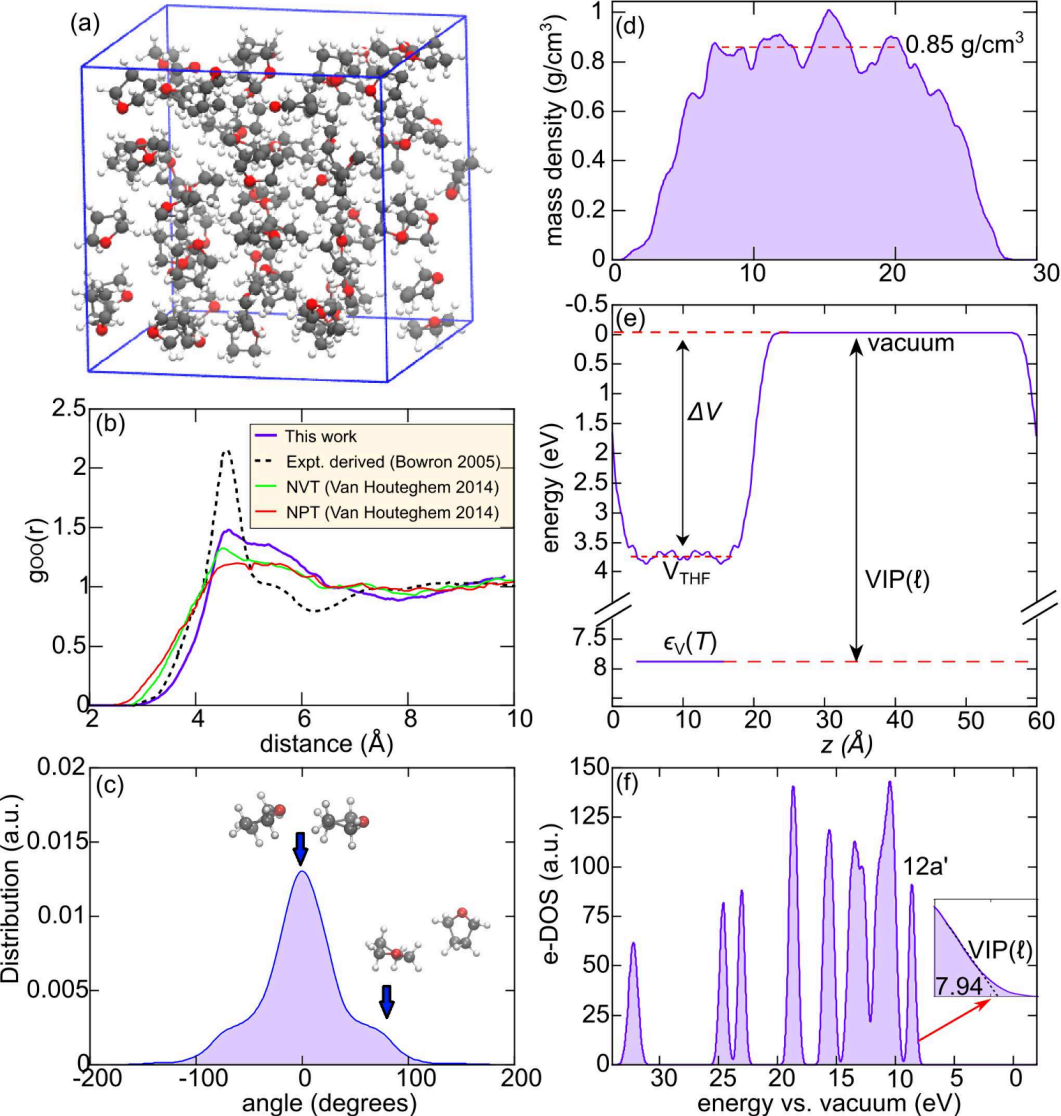
(a) Stick and ball representation of the cubic supercell employed to simulate l -THF (C atoms in gray, O in red, H in white). (b) Oxygen–Oxygen radial distribution function for l -THF from the present MD simulation, compared with previous experimental and computational studies.^58,59^ (c) Distribution of angles formed by the molecular planes of first-neighbor THF pairs, using oxygen atoms as reference points. (d) Planar averaged mass density of l -THF at the interface with vacuum. (e) Schematic representation of the alignment at the l -THF/vacuum interface for the valence band edge. (f) Averaged electronic density of states calculated for l -THF. Energies are referred to the vacuum level. We highlight the position of the peak associated with the 12a′ molecular orbital of THF, and in the inset, we show the linear extrapolation employed to evaluate the vertical ionization potential of l -THF, VIP­( l ).

We further investigate the microscopic structure of l -THF by inspecting the relative orientation of THF molecules in the liquid. To this end, we compute the angular distribution between the molecular planes of interacting pairs, using the oxygen atoms as reference points. The distribution in Figure 1 (c) shows a clear peak centered at 0°, suggesting a preferred parallel orientation of the THF molecules, i.e. essentially lying on the same plane or stacked on top of each other. Two pronounced shoulders peaking at ≈ ± 74° are related with THF molecules arranged in an almost perpendicular fashion, see inset of Figure 1 (c) for representative MD snapshots. Such a result is consistent with DFT calculations indicating the parallel arrangement of THF dimers to be the most energetically favorable, but with differences below 0.1 eV with respect to other configurations, cf. ref 60 and SI, section S4.

Since energy levels obtained from periodic supercell calculations are not aligned with respect to a physical reference, we perform an additional MD simulation of the atomistic l -THF/vacuum interface. In this way, we can refer the electronic structure of l -THF with respect to the vacuum level and estimate the absolute energy levels of l -THF. To this end, we expand the simulation cell used to model the bulk in one direction to include 40 Å of vacuum (*a* = *b* = 20 Å and *c* = 60 Å) and evolve the MD further for 2 ns. We monitor the averaged mass density of l -THF in the interior region: it amounts to 0.85 g/cm^3^ [cf. Figure 1 (d)], a value differing from that of the bulk by only 0.03 g/cm^3^, thus being irrelevant for the subsequent calculation of the potential offset at the interface with vacuum.

We then perform electronic-structure calculations at the hybrid DFT theory level on top of a set of 100 MD structural configurations equally spaced in time (one each 10 ps of simulation) for (i) the bulk l -THF system to assess the energy levels and (ii) the l -THF/vacuum slab to calculate the potential offset Δ*V*_int_ across the interface. We note that, with this procedure, we achieve average values with statistical errors below 0.05 eV for all the quantities presented in this study, as estimated from blocking analysis (cf. SI, Section S3 for an example).^61^ We adopt the KC-DFT method derived from the PBE0 hybrid functional,^62,63^ namely PBE­(α_K_), which has shown to deliver electronic properties in excellent agreement with the experiment for a plethora of condensed-phase systems.^45,47,64–67^ The fraction of Fock exchange α to be included in the functional is determined via the probe method^47,65,66^ and is found to be 52%. Nonlocal dispersion interactions are included via the self-consistent rVV10 method.^68,69^ All the calculations are performed with the CP2K suite of programs^70–74^ (cf. Section S1 of SI for computational details and S4 for tests of the accuracy of the methods for gas-phase systems and THF dimers).

We first discuss the potential offset Δ*V* across the interface, i.e the difference between the average electrostatic potential in the bulk-like region of THF in the slab, *V*_THF_, and the vacuum level. While we find a residual electric field in the vacuum region of only 0.003 eV/Å from our calculations, we nevertheless eliminate the bias arising from it by symmetrizing the potential profile.^75^ We estimate a value of 3.73 eV, cf. Figure 1 (e), which allows for a direct alignment of the energy levels of l -THF, as calculated from supercells of the bulk system, namely the valence and conduction band edges at room temperature, ϵ_V_(*T*) and ϵ_C_(*T*), respectively. These are evaluated via linear extrapolation of the wing of the density of states, in analogy with the experimental characterization of liquids (e.g., water in refs 76, 77 ) and are devoid of size-dependent effects of the band tail influencing the estimate of these quantities from MD simulations.^67,78^

In Figure 1 (f), we illustrate the averaged electronic density of states (e-DOS) for l -THF, aligned with respect to the vacuum level. We find the vertical ionization potential of l -THF, VIP­( l ), to lie 7.94 eV below the vacuum level, deviating by only 0.14 eV from the experimental value of ≈ 7.8 eV inferred from the photoemission spectrum of ref 54 (cf. Table 1). The width of the experimental spectrum is also nicely reproduced: the main peak of the valence band edge, related to the mixing of the 12a’ molecular orbitals of the THF molecule,^79^ is found at 8.63 eV below the vacuum level, very close to the measured value of 8.5 ± 0.1 eV.^54^ The vertical electron affinity, VEA­( l ), of the system is found at 1.43 eV above the vacuum level: this, to the best of our knowledge, represents the *first* estimate of this quantity in the condensed phase and it aligns with the general behavior of saturated organic molecules, which typically do not favor the addition of an extra electron.^80^ In Table 1, we also report the calculated and measured gas-phase values for the vertical ionization potential of THF, VIP­(*g*). Our theoretical estimate at 9.70 eV (achieved from the lowest-energy conformer of the molecule, cf. Figure S2 and Table S2) is in striking agreement with the average literature value of 9.71.^79^

**Table 1:** Calculated and Experimental Electronic Levels of THF for the Gas and Liquid Phases^a^

	VIP(*g*)	VIP( l )	12*a*′ peak ( l )	VEA( l )
Theory	9.70	7.94	8.63	–1.20
Expt.	9.71 ± 0.03^a^	7.80^b^	8.5 ± 0.1^b^	//

a All values are given in eV.

b Photoelectron spectra of THF from ref 79

c Photoemission spectroscopy measurements from ref 54

Having demonstrated that the structural and electronic properties of l -THF are reasonably reproduced, we next model its solution with NAP. We first consider the neutral system, which is modeled by replacing a single THF molecule in the simulation cell with a NAP molecule, and we then perform another MD simulation (cf. SI, section S1 for details).

Inspection of the highest occupied and lowest unoccupied molecular orbitals as well as the averaged e-DOS for the neutral NAP/ l -THF system, cf. Figure 2 and S6, indicates that the insertion of the solute brings to both occupied and empty localized states within the energy gap of the liquid. The position of the peak associated with the highest occupied molecular orbital (HOMO) of NAP in the averaged e-DOS allows us to position its VIP at 7.07 eV vs vacuum, differing only by 0.11 eV from the measured value^54^ (cf. Table 2), a satisfying result achieved thanks to the correct description of the l -THF band edges at the hybrid KC-DFT level.^81,82^ Again, for the sake of comparison, we also compute the gas-phase VIP of NAP, which is found at 8.05 eV vs vacuum, in excellent agreement with the most recent measurements,^83^ see Table 2.

**Figure 2:**
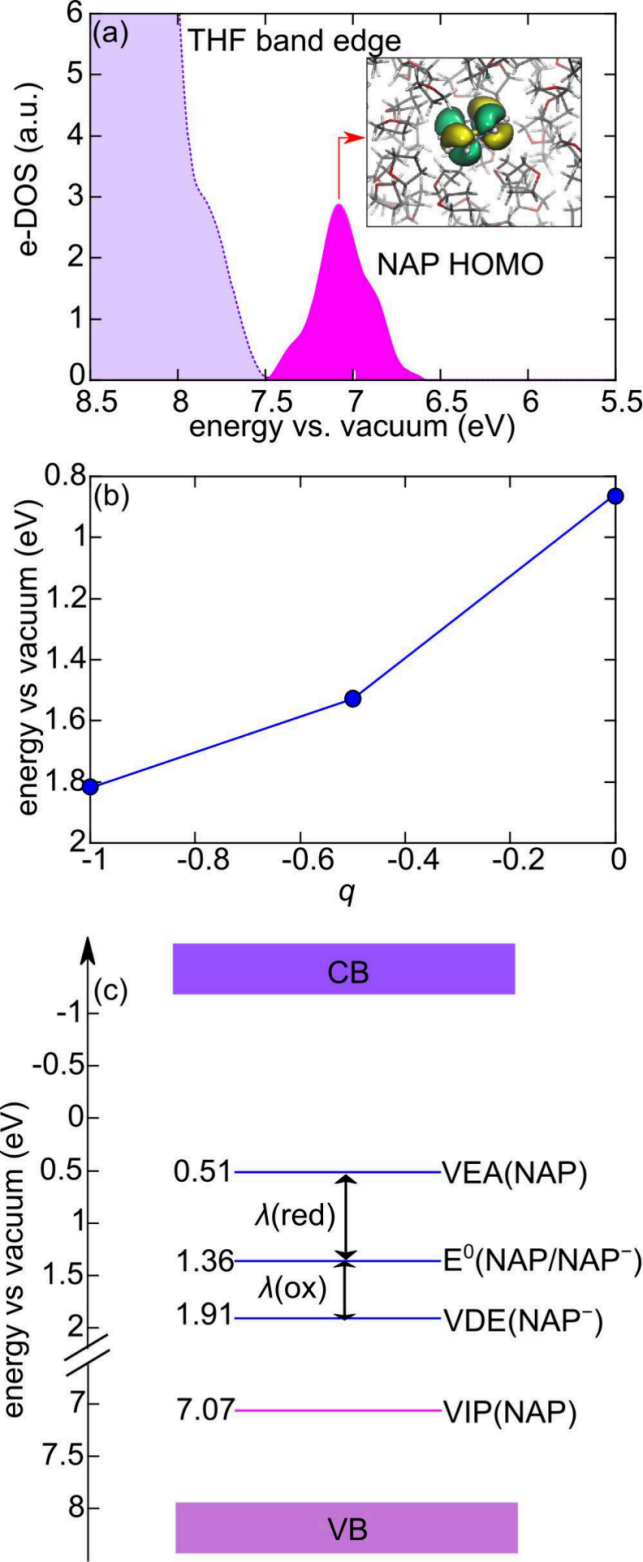
(a) Electronic density of states (e-DOS) of the occupied energy levels for the l -THF/NAP atomistic model. The peak relative to the highest occupied molecular orbital of NAP is given in magenta. The isodensity representation (isovalue 0.01) of the HOMO for a structural configuration is shown in the inset. (b) ⟨Δ*E*⟩_*q*_ (cf. main text for definition) as a function of the charge *q*. Values are referred with respect to the vacuum level. (c) Schematic representation of the energy levels associated with the NAP/NAP^–^ redox couple in l -THF.

**Table 2:** Calculated and Measured Electronic Energy Levels and Reorganization Energies for the NAP/NAP^–^ Redox Couple in the Gas Phase and in THF Solution

Gas phase	This work	Expt.
VIP(NAP)	8.05	8.15^a^
VEA(NAP)	–0.50	//
VDE(NAP^–^)	–0.20	–0.18^b^
*E*^0^(NAP/NAP−)	–0.35	–0.19^c^
λ_int_	0.14	//

THF Solution	This work	Expt.
VIP(NAP)	7.07	7.18^d^
VEA(NAP)	0.51	//
VDE(NAP^–^)	1.91	2.73^e^
*E*^0^(NAP/NAP−)	1.36	2.05^f^-2.21^e^
λ(red)	0.85	//
λ(ox)	0.55	0.52–0.68^g^

a Photoemission spectrum of gas-phase NAP from ref 83.

b Value extrapolated from photoemission measurements performed on clusters in refs 93–95 .

c Inferred from electron transmission spectra of ref 96

d Photoemission spectrum of NAP in THF solution from ref 54

e Photoemission spectra of NAP^–^ in THF solution from ref 54, value referred to the ion pair with a metal cation

f Voltammetry measurements from ref 97 for the NAP^–^/metal cation pair

g Difference between VDE of ref 54 and available *E* ^0^ estimates, cf. eq 10.

Then, since our main objective is to determine the vertical and adiabatic redox potentials associated with the NAP/NAP^–^ couple and then infer the external reorganization energy from them, we adopt the grand-canonical formulation of solutes in solution mimicked from that of defects in semiconductors^84,85^ and originally developed for the aqueous environment.^78,86,87^ Within this approach, the redox potential associated with the following half-reaction:

1 $$NAP\left(THF\right)+e^{-}\to {NAP}^{-}\left(THF\right)$$

is defined as^78,87^

2 $$E^{0}\left(NAP/{NAP}^{-},THF\;vs.vacuum\right)=\Delta G_{red}-E_{corr}\left(-,R_{-1}\right)$$

where:

3 $$\Delta G_{red}=G\left(-1,R_{-1}\right)-G\left(0,R_{0}\right)$$

In eq 2, Δ*G*_red_ = *G*(−1, *R*_–1_) – *G*(0, *R*_0_) is the free energy difference between the anion and the neutral solute in their respective equilibrium structures R_*q*_. This term is first referred to ϵ_V_(*T*) and then aligned with respect to the vacuum level via the potential offset, see Figure 1 (e). *E*_corr_(−,*R*_–1_) is a term included to correct the electrostatic finite-size error, typical of DFT calculations including a solute/defect with charge *q* and coordinates *R*_*q*_ in a periodic supercell (cf. refs 84, 85, 88 and section S1 of SI for details on the correction). Δ*G*_red_ is here calculated employing thermodynamic integration of vertical energy gaps:^89^

4 $$\Delta G_{red}=\int_{-1}^{0}{\left\langle \Delta E\right\rangle }_{q}dq$$

where ⟨Δ*E*⟩_*q*_ is the vertical energy gap between neutral and negatively charged solute in the condensed phase. This is calculated on the structural configurations from the MD simulations at charge *q*:

5 $${\left\langle \Delta E\right\rangle }_{q}=E\left(-1,R_{q}\right)-E\left(0,R_{q}\right)$$

In this study, we consider the neutral solute (*q* = 0), its anion (*q* = – 1) as well as a fictitious system in which a fractional charge of 0.5 *e* ^–^ is imposed on NAP. This three-point approximation to the thermodynamic integral is used to account for deviations from the linear Marcus regime typically encountered for redox potentials in solution^78,90^ and represents an excellent compromise between accuracy and computational cost, if compared with free-energy calculations with higher number of intermediate points between reactants and products.^91,92^ For calculations including an unpaired electron, the unrestricted Kohn–Sham formalism is adopted. All the vertical energy gaps are again averaged over calculations on top of 100 MD configurations equally spaced in time (one each 10 ps). ⟨Δ*E*⟩_*q*_ values show a slight deviation from linearity, cf. Figure 2 (b), which leads to a difference of 0.15 eV in the calculated redox level with respect to the Marcus approximation, as thermodynamic integration gives *E* ^0^(NAP/NAP^–^, THFvsvacuum) = 1.36 eV.

The vertical electron affinity of NAP, VEA­(NAP, THF), and the vertical detachment energy of NAP^–^, VDE­(NAP^–^, THF), are defined, respectively, as follows:

6 $$VEA\left(NAP,THF\right)={\left\langle \Delta E\right\rangle }_{0}+E_{corr}\left(-1,R_{0}\right)$$

7 $$VDE\left({NAP}^{-},THF\right)={\left\langle \Delta E\right\rangle }_{-1}-E_{corr}\left(0,R_{-1}\right)+E_{corr}\left(-1,R_{-1}\right)$$

In eqs 6 and 7, *E*_corr_(−1, *R*_0_) is an electrostatic finite-size term necessary to correct the total energy of the negatively charged periodic supercell upon vertical injection of an electron into the neutral system (*R*_0_), while *E*_corr_(0, *R*_–1_) is the correction to the energy of the neutral system formed when vertically detaching an *e* ^–^ from the anion (*R*_–1_) in the periodic cell (see ref 88 and Section S1 of SI for details). The complete alignment is illustrated in Figure 2 (c). In Table 2, we also collect, for comparison, the available experimental data in solution, as well as calculated and measured gas-phase data.

We first comment on the electron affinities in the gas phase, as these are known to be more challenging for computational methods than ionization potentials^98,99^ and, in this case, even experimental values for vertical and adiabatic energy levels should be taken with caution. In fact, on one side, it is claimed that the value of – 0.19 eV from electron transmission spectra is adiabatic, notwithstanding the vertical nature of this technique.^96^ On the other hand, VDEs between – 0.18 eV and – 0.20 eV have been achieved from extrapolation of PES measurements performed on cluster data.^93–95^ The combination of these data would imply a vanishingly small internal reorganization energy of the molecule, defined as

8 $$\lambda_{\mathrm{int}}=VDE\left({NAP}^{-},g\right)-E^{0}\left(NAP/{NAP}^{-},g\right)$$

We here calculate *E* ^0^(NAP/NAP^–^, *g*) = −0.35 eV and VDE­(NAP^–^, *g*) = −0.20 eV, values well within the experimental range reported in literature. The resulting λ_int_ = 0.15 eV is in good agreement with previous computational studies based on either many-body perturbation theory or the coupled cluster family of methods,^99–102^ thus reassuring us of the robustness of our approach.

When focusing on the redox levels in solution, we calculate *E* ^0^(NAP/NAP^–^, THF vs vacuum) = 1.36 eV, thus observing differences up 0.8 eV with respect to the experiment. However, we underline that, in ref 54, solvated NAP^–^ is produced via addition of alkali metals to the solution. Hence, the energy levels, inferred from photoemission spectroscopy, do not correspond to those of the free ion but rather to a ion pair formed with the metal cation.^54^ Analogous experimental set-ups have been adopted in electrochemical measurements, giving similar results.^97^ Therefore, we can ascribe the observed difference to this effect: the lower values of vertical and adiabatic levels in the experiment imply enhanced stabilization induced by Coulomb interactions. We note that, when using the PCM^15^ method to calculate *E* ^0^(NAP/NAP^–^, THF vs vacuum), we obtain a value of 1.45 eV, within 0.1 eV with the MD-achieved results (cf. section S1 of SI for details). This confirms that the difference with the experiment actually originates mainly from the ion pairing occurring in the latter. We further pinpoint that our choice of considering the free ion is motivated by the fact that, in ET experiments, organic radical anions, such as NAP^–^, are usually generated by pulse radiolysis, a technique that disfavors ion pairs.^51^ For such systems, we aim at evaluating the external reorganization energy in the following.

Due to nonlinear solvent response, evidenced by the thermodynamic integration, we define two total reorganization energies, one associated with the reduction of NAP, λ­(red), and another to the oxidation of NAP^–^, λ­(ox), respectively [cf. Figure 2 (c)]:

9 $$\lambda \left(red\right)=E^{0}\left(NAP/{NAP}^{-},THF\right)-VEA\left(NAP,THF\right)$$

10 $$\lambda \left(ox\right)=VDE\left({NAP}^{-},THF\right)-E^{0}\left(NAP/{NAP}^{-},THF\right)$$

Since λ = λ_int_ + λ_ext_, we can estimate the respective solvent reorganization energies, employing, as λ_int_, the value here calculated for the gas-phase molecule, which is within 0.03 eV of those previously obtained with structural optimizations in implicit THF solvent.^6,7^ Here we infer λ_ext_(red) = 0.70 eV and λ_ext_(ox) = 0.40. For comparison, we also list in Table 3 (i) the values inferred from our simulations, considering Marcus approximation, and (ii) those calculated employing the nonequilibrium PCM method (NEPCM).^103^ As Marcus approximation and the implicit solvent cannot capture nonlinear solvent response, we achieve equivalent reorganization values for reduction and oxidation processes. In particular, NEPCM tends to overestimate solvent reorganization energies, due to the neglect of explicit solute–solvent interactions,^15^ with differences up to 0.42 eV in the present case.

**Table 3:** External Reorganization Energies, Estimated from the Redox Energy Levels Calculated Using the Thermodynamic Integration (TI) Method and the Marcus Approximation (Linear Solvent Response)^a^

	λ_ext_(red)	λ_ext_(ox)
This work (TI)	0.70	0.40
This work (Marcus)	0.55	0.55
NEPCM	0.82	0.82

a For comparison, we also report the values calculated using an implicit solvent model, namely the NEPCM method. All values are given in eV.

We note that, when reorganization energies enter kinetic models of ET, they usually include both the donor and the acceptor contribution. For the outer-sphere self-exchange ET of NAP anion in solution we would compute an external reorganization energy amounting to 1.1 eV, which is of the same order of magnitude as evaluated for other ET reactions in THF in ref 7, using both the original Marcus expression,^2,3^ and the SOLVMOL package based on the nonlocal response theory proposed by Matyushov.^104^ These values have been calculated considering different donor and acceptor molecules separated by a large androstane unit. While the data from ref 7 are not directly and quantitatively comparable with the isolated molecule in solution considered here, they still indicate that the total λ_ext_ can be sizable even in a moderately polar solvent, such as THF. We finally remark that the Marcus approximation could still give numerically valid estimates of the total λ_ext_, provided that donor and acceptor molecules entail a similar symmetry/asymmetry in solvent response, while it may severely fail if the interactions of the donor and the acceptor with the solvent are sensitively different. Anyway, this approximation cannot capture all the essential physical interactions underlying the calculated values of λ.

To gain further insights into the microscopic origin of the solvent motion related with the reorganization energy, we analyze the solute–solvent interactions ensuing from our MD simulations of solvated NAP and NAP^–^. We first inspect the RDFs for the center of mass (CM) of NAP and the oxygen atoms of THF. For the neutral solute, we observe a peak at 5.67 Å with a long shoulder, up to 8.50 Å, indicating a substantial degree of disorder and weak interactions. When considering NAP^–^, we immediately notice a sizable shift of the peak from 5.67 to 6.37 Å, while the size of the coordination shell is preserved. This clearly indicates a solvent motion induced by the negative charge on the NAP^–^, which translates into a rotation of THF molecules in the first solvation shell, pointing their partially negative oxygen atoms away from the electron density.

We then consider the RDF for the CMs of both solute and solvent molecules, cf. Figure 3 (b). The most prominent difference between the neutral solute and the anion is the appearance, for the latter, of a clear shoulder in the first solvation shell at a distance 1.5 Å shorter than that of the main peak. This is somewhat surprising since a mere displacement of O atoms due to electrostatic repulsion should produce only a minuscule shift of the main peak toward higher distances, which we indeed observe. Instead, the presence of an additional shoulder at shorter distances, including, on average, 1.25 THF molecules, denotes the presence of a weak but clear inductive effect. This is related to loosely bound solvent molecules, polarized by the electric field of the anion and becoming closer to it. We roughly estimate the effect of this local interaction with a NAP-THF dimer model and find it to be comparable to other contributions to the reorganization energy, cf. section S7 of SI. Analysis of the spin density for configurations belonging to the shoulder indicates that it does not protrude toward the closest THF molecule but we observe a slight asymmetry, cf. Figure S7. This is confirmed by analysis of the Mulliken spin population, which reveals that one of the NAP rings accounts for 58% of the spin density, thus denoting a slight polarization of the solute.

**Figure 3:**
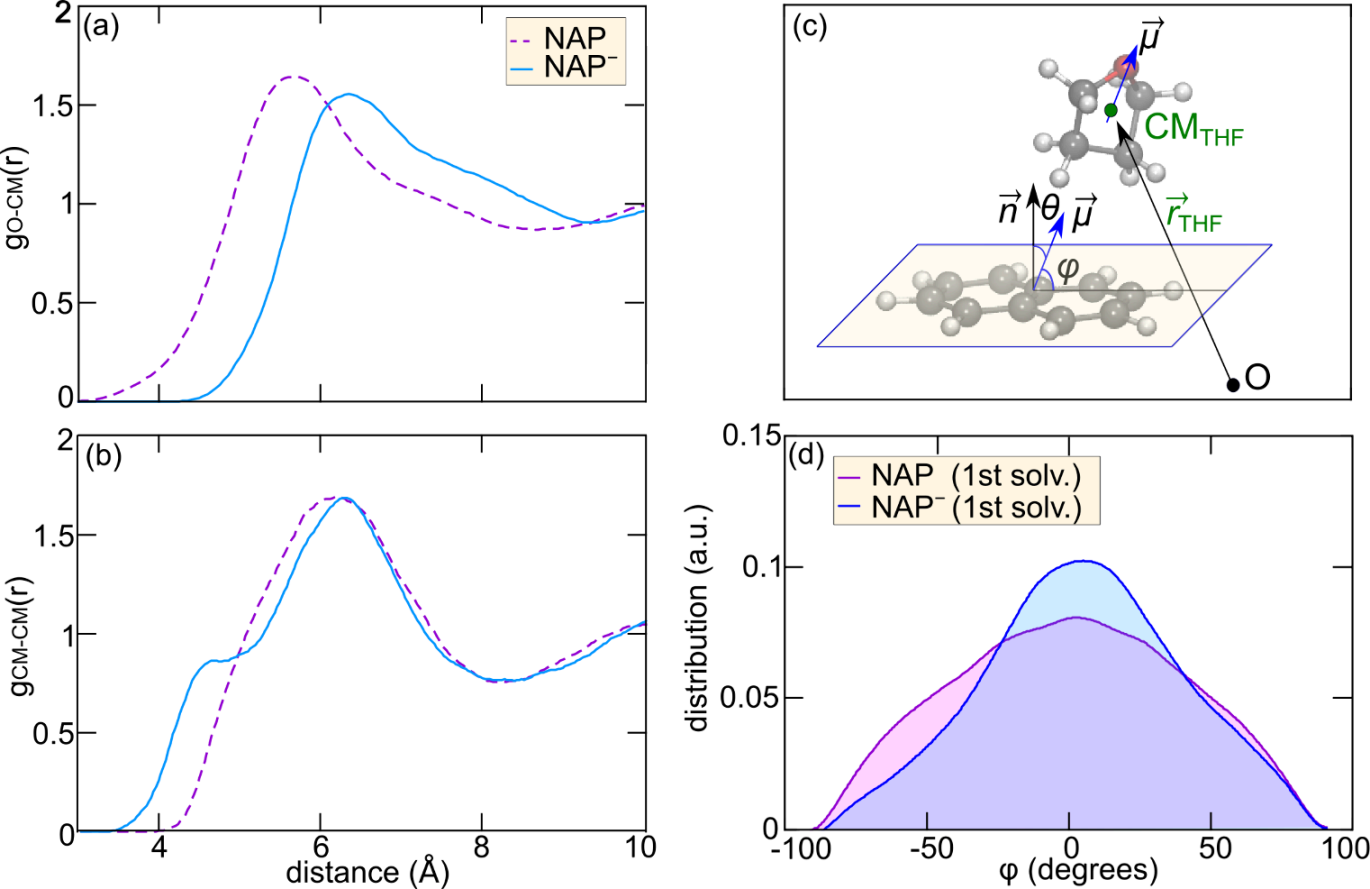
Radial distribution function between (a) the center of mass (CM) of the solute and the O atoms of THF for NAP and (b) the center of mass of the solute and solvent molecules for NAP and NAP^–^ in l -THF. (c) Graphical representation of the ϕ angle between the solute molecular plane and the THF dipole moment. (d) Distribution of the angle ϕ between the solute molecular plane and the THF electric dipole moment for neutral NAP and its anion, as calculated for the first solvation shells, evaluated from the respective MD simulations.

The dipolar contributions to the calculated external reorganization energy can be understood by observing the orientation of the THF dipoles with respect to the molecular plane of the solute for NAP and NAP^–^ solutions. To this end, we define the angle ϕ, between the THF dipole moment and the NAP molecular plane, cf. Figure 3 (c). This is the complementary of θ, i.e. the angle between the normal vector to the plane, n⃗, and the THF molecular dipole μ⃗. For details on the way in which all these quantities has been evaluated, see section S8 of the SI. Within the definition of the angle, positive (negative) values refer to dipoles pointing away (toward) the molecular plane.

We calculate the distribution of the ϕ angle considering the full first solvation shell, defined by the minimum of the CM-CM RDF and enclosing ≈ 15 THF molecules (d). The distribution, centered almost on 0° and essentially symmetric for the neutral solute, becomes asymmetric and its peak shifts to a positive angle for the anion. The asymmetry in the distribution appears to be even more pronounced if we limit the calculations to a progressively smaller subset of the first neighbors, for which a clearer shift toward more positive angles is observed, i.e. molecular dipoles pointing away from the charged solute, cf. Figure S9. This again is in accordance with a physical interpretation that sees the motion of only a few solvent molecules as crucial in the response upon charge injection.^29–31^

Overall, the present results allow for an interpretation of the observed nonlinear solvent response: in fact, the smaller value of λ_ext_(ox) originates from the fact that the orientation (and the distance) of THF molecules in the first solvation shell is fundamental in stabilizing the charged system, while it is less relevant upon electron detachment, when the solute goes back to its neutral (and apolar) state, with a less ordered microsolvation. Further, by monitoring the time-dependent evolution of the reorganization, we observe that reorganization upon vertical injection (detachment) is carried out within ≈ 10 (≈ 100) ps (section S10 of SI). These time-scales comparable to those of THF rotational motion^105,106^ and the differences are again consistent with the dissimilar nature of the interactions leading to reorganization and with nonlinear solvent response. In a schematic picture, we can say that the parabolas describing the free-energy profile as a function of the solvent polarization have a different curvature, as schematized in Figure 4.

**Figure 4:**
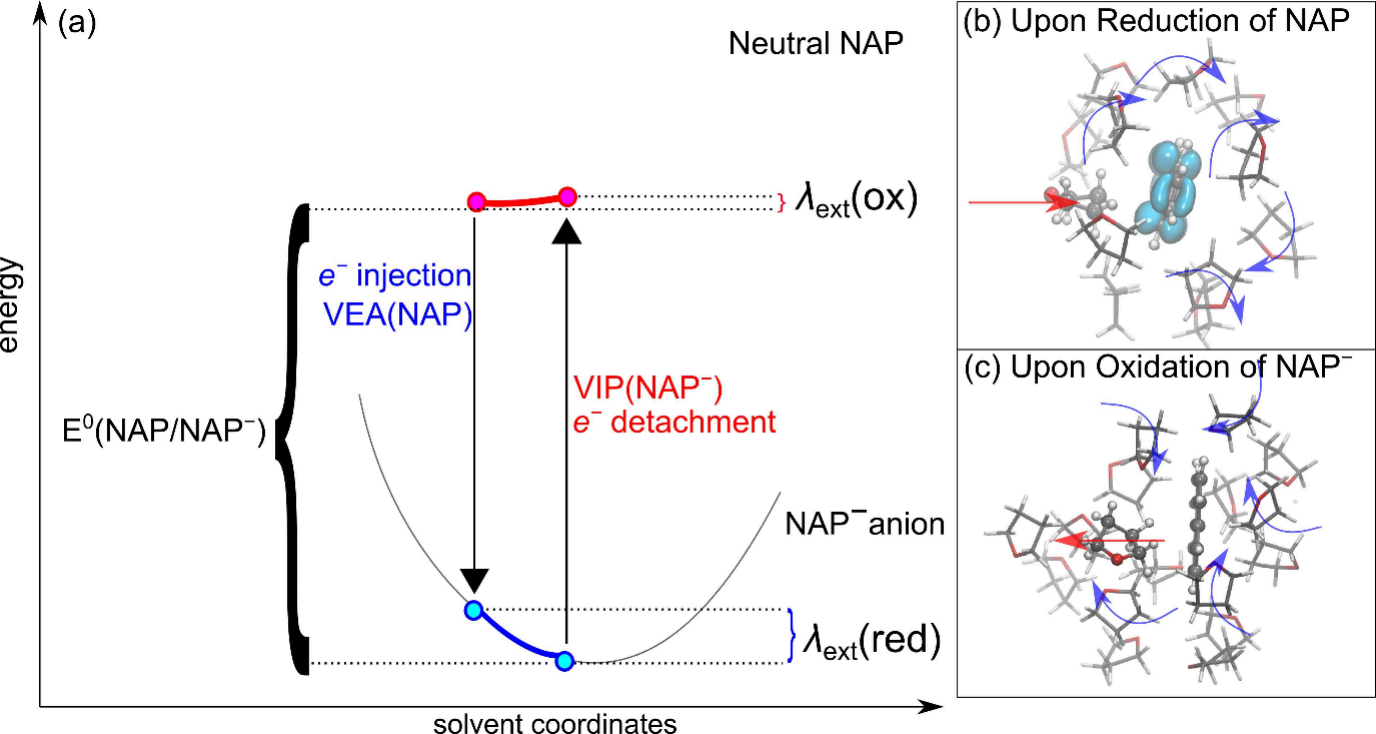
(a) Schematic representation of the asymmetry in solvent reorganization energies upon reduction of NAP and oxidation of NAP^–^. The relevant vertical and adiabatic redox levels are also reported for clarity. Stick and ball representation of a representative structural configuration for the first solvation shell for (b) NAP and (c) NAP^–^ in THF solution. The different contributions to the external reorganization energy, as discussed in the main text, are also reported.

Furthermore, we find that, while dipole-charge interactions represent an important driving force for the reorganization upon variation of the solute’s charge state, effects associated with induced polarization, which dominate the solvent response in apolar environments,^9,36,40^ still play a significant role also in a mildly polar solvent, such as l -THF.^30,31^ We remark that the standard Marcus approach incorporates the effect of the solute on λ_ext_ simply via the molecular radius. However, the local interactions observed here are likely to be extremely solute-dependent and the addition of electron-withdrawing/donating groups to a molecule may dramatically alter the microsolvation, even if the size of the solute is basically unvaried.

When reviewing the present results in the context of the current literature, which is mainly dedicated to aqueous solutions, we observe that both linear and nonlinear solvent responses have been characterized, depending on the nature of the reactants.^107^ For example, the charge transfer reactions related to Ru^3+^/Ru^2+^ redox couple were found to display a frankly linear response.^24^ In stark contrast, other systems, e.g. OH·/OH^–^ and CO_2_^‑^/CO_2_ couples, showed a remarkable nonlinear behavior.^78,108^ In THF, it has been shown, by both theory and experiment,^29,109^ that equilibrium solvation of Na is achieved more rapidly from vertical reduction of Na^+^ than from vertical ionization of Na^–^, a finding which demonstrates a breakdown of linear response. Overall, these results suggests that the diversity in solute–solvent reactions upon charge transfer, which is substantial when considering a redox couple formed by a neutral and a charged solute, surely contributes to asymmetry in external reorganization energies also in mildly polar solvents.

In conclusion, we investigated the electronic energy levels of l -THF and its solutions with NAP and NAP^–^, employing a combined approach including classical MD simulations and hybrid-DFT calculations. Our methodology provides an accurate and computationally efficient framework to study solvent-induced effects on electronic levels and evaluate solvent reorganization energies. We demonstrated that explicit solvent modeling captures both dipole-charge interactions and induced polarization effects, which are crucial in moderately polar solvents like l -THF. These findings highlight the limitations of continuum solvation models in accurately describing solvent reorganization and emphasize the necessity of atomistic simulations for a more realistic picture of solute–solvent interactions, particularly when dealing with the delicate balance of different interactions, which is relevant for mildly polar solvents. Beyond the specific case of naphthalene in THF, our approach, combining fast and accurate classical dynamics with advanced electronic-structure calculations, represents a powerful and portable tool for systematically investigating solvation effects in a variety of solvents and solutes, including more complex charge transfer processes. Therefore, the natural continuation of this will be (i) extending the methodology to a wider range of solvents and solutes, further refining our understanding of solvent-dependent electronic properties and their implications for ET dynamics in the condensed phase, and (ii) estimating the ET rates for complete donor–acceptor systems.

## Supplementary Material



## Notes

Computational details of molecular dynamics simulations and of the electronic-structure calculations at the KC-DFT level, along with supplementary tests on the functional. Additional references (PDF). Input files, models, and snapshots of MD simulations are available at https://github.com/XelaleX1/Reorg_THF_paper.
