# Supplementary material for: External Reorganization Energy upon Charge Transfer Reactions in Mildly Polar Media: The Case of Naphthalene in Tetrahydrofuran
Source: J Phys Chem Lett. 2025 Jun 24;16(26):6734–44. doi: 10.1021/acs.jpclett.5c01328 (PMC12235641; doi:10.1021/acs.jpclett.5c01328)
Supplement: Supplementary file 1 [file jz5c01328_si_001.pdf]

Supplementary Information for: External Reorganization Energy upon Charge Transfer Reactions in Mildly Polar Media: The Case of Naphthalene in Tetrahydrofuran

Francesco Ambrosio,*,†,‡ Alessandro Landi,*,‡ Michele Loriso,† Anna Leo,‡ and Andrea Peluso‡

†*Dipartimento di Scienze di Base e Applicate (DISBA), Università degli Studi della Basilicata, Viale dell'Ateneo Lucano, 10 - 85100 Potenza, Italy*

‡*Dipartimento di Chimica e Biologia Adolfo Zambelli, Università di Salerno, Via Giovanni Paolo II, I-84084 Fisciano (SA), Italy*

E-mail: francesco.ambrosio@unibas.it; alelandi1@unisa.it

S1 Computational Details

Classical Molecular Dynamics

Classical molecular dynamics (MD) with QM-derived force fields provide results in good agreement with experiments[1–3] and consistent with more expensive DFT-based molecular dynamics (DFT-MD) simulations.[4] For these reasons, we have here alleviated the computational burden by performing single-point DFT calculations on top of snapshots extracted from classical MD simulations. We note that using a DFT functional based on the generalized gradient approximation (GGA) might not only be impractical to generate long trajectories but may also lead to unphysical dynamics when performing MD simulations on systems with an unpaired number of electrons, a consequence of the self-interaction error.[5,6] The incorrect description of the energy gap of the liquid might impede charge localization on the solute, an instance which has been verified several times, e.g. in liquid water, see Refs. 5,6. Further, we note that tight-binding methods, although computationally cheaper than full DFT, remain significantly more expensive than classical MD and would still require subsequent re-evaluation of energies and electronic properties at a higher level of theory to reach the accuracy needed in this work. This would largely negate the computational advantage. Finally, another possible choice would have been machine-learned potentials. These, while promising, would necessitate the creation of a dedicated and carefully curated training set for each specific solvent and solute system. In our case, this added complexity is unnecessary: the QMD-FF parameterization protocol we employ is fully automated, transferable, and delivers excellent agreement with experimental and high-level theoretical benchmarks. Thus, our approach strikes a favorable balance between computational efficiency and accuracy, particularly well suited to the study of solvent reorganization in weakly polar environments.

We first produce accurate intramolecular quantum-mechanically-derived force field (QMD-FF) parameterization for all the molecules under study (for NAP, we considered both the neutral molecule and the radical anion), adopting the Joyce parameterization protocol,[1,7,8]

which exploits a fitting procedure of the Hessian matrix obtained at the B3LYP[9]/6-31G(d) level of theory using the Gaussian16 software.[10] The description of the molecules for classical MD simulations has been completed by combining the intramolecular QMD-FFs with Lennard-Jones parameters from appropriate OPLS (Optimized Potentials for Liquid Simulations) atom types,[11] and point charges fitted following the RESP (Restrained Electrostatic Potential) procedure.[12] The FF for the fictitious system, in which a fractional charge of 0.5 e^- is imposed on NAP, has been obtained from the neutral one, but putting the charges as equal to the average between the neutral and the charged FFs. The FFs have been made available to the community in public GitHub repository.

Using these QMD-FFs, we carry out classical MD simulations with the GROMACS 2020.5 software.[13] The supercell adopted to simulate ℓ-THF has a cubic shape with a = 20.0 Å and contains 60 THF molecules. When NAP is inserted, it is placed in the center of the supercell and one THF molecule is removed to accommodate the solute and preserve solvent density. We include periodic boundary conditions and take into account long-range electrostatic effects through the PME algorithm.[14] We use an integration time step of 2 fs, imposing constraints on the bonds involving H atoms through the LINCS algorithm,[15] adopting a modified Berendsen thermostat[16] to control temperature and a Berendsen barostat to control pressure[16] (for NPT simulations). For each MD simulation, we perform an initial steepest descent minimization, followed by a slow NVT heating procedure to 298 K. After equilibrating the system at the target temperature in NVT conditions for 0.5 ns, we carry out production runs in NPT conditions for ℓ-THF and in NVT conditions for the solutions with NAP. MD runs last for 2 ns.

Density Functional Theory Calculations on Supercells

In order to accurately evaluate the energetics and the electronic properties from the MD structural configurations achieved for the atomistic models of ℓ-THF, its interface with vacuum and solutions with NAP and NAP$^-$, we require a DFT level of theory, in which the self-

interaction error, typical of standard methods, has been silenced. To this end, we construct a Koopman's compliant (KC) hybrid functional for the host system, i.e. ℓ-THF, adopting the simplest hybrid-DFT scheme, based on the PBE0 family of functionals.[17,18] The probe method[19–21] is used to determine the fraction of Fock exchange fulfilling the generalized Koopmans' condition, α_K. We insert a fluorine atom in a MD-structural configuration of the atomistic model for ℓ-THF, thus introducing a localized state in the energy gap of the liquid. Then, we calculate the single-particle energy level for the neutral system (F) and for the negatively charged system upon vertical injection of an electron (F^-) at the PBE(α) level of theory, with different values α. Then, α_K is defined by the intersection between the linear evolution of the single-particle energy levels for the neutral and charged system, as done in previous studies on other liquid systems such as water[21] and dichloromethane.[22] This procedure gives $\alpha_K = 52\%$. This level of theory complemented with non-local electron correlation described at the rVV10 level[23,24] is used for all supercell calculations presented in this study. The b parameter governing the extent of non-local correlation is kept to its original value of 6.3.[23,24] We note that, while rVV10 still relies on a phenomenological formulation, once the parameters are set, the resulting functional can be carried over to any weakly bonded system without requiring any further parameter tuning. The complete exchange-correlation functional used in this work reads as follows:[25]

$$E_{\mathrm{xc}} = \alpha_K E_{\mathrm{x}}^{\mathrm{F,TC}}(R_c) + \alpha_K E_{\mathrm{x}}^{\mathrm{PBE,LRC}}(R_c) + (1-\alpha_K)E_{\mathrm{x}}^{\mathrm{PBE}} + E_{\mathrm{c}}^{\mathrm{PBE}} + E_{\mathrm{c}}^{\mathrm{nl}}. \tag{S1}$$

In Equation S1, $E_{\mathrm{x}}^{\mathrm{F,TC}}(R_c) + E_{\mathrm{x}}^{\mathrm{PBE,LRC}}(R_c)$ is the range-separated exchange energy,[26] including the Fock exchange with the truncated Coulomb (TC) operator and the long-range correction (LRC) based on the PBE exchange hole. A cut-off radius R_c of 7 Å is here considered for the calculation of the exchange integrals. $E_{\mathrm{x}}^{\mathrm{PBE}}$ is the PBE exchange, $E_{\mathrm{c}}^{\mathrm{PBE}}$ and$E_{\mathrm{c}}^{\mathrm{nl}}$ are the PBE and non-local correlation terms, the latter being calculated with the rVV10 formulation.

Supercell calculations have been performed using the freely available CP2K\QUICKSTEP

package.[27] In this code, core electrons are treated via analytical Goedecker-Teter-Hutter pseudopotentials,[28] while valence electrons are described with atomic basis sets. In particular, we use the MOLOPT double-zeta polarized basis set[29] and a cutoff of 800 Ry for the plane waves. CP2K\QUICKSTEP allows for high-throughput hybrid-DFT calculations, in virtue of the implemented auxiliary density matrix method (ADMM), which limits the expansive calculation of two-electron integrals to an auxiliary basis set. In particular, we adopt the cFIT3 basis set, developed by Guidon et al.[30,31], which we found to be sufficient to achieve accurate total energy differences and orbital energies (vide infra). The complete input file is available in public GitHub repository.

The definition of localized energy levels within the energy gap of a solid or a liquid usually is not dependent on the fraction of Fock exchange included in the hybrid functional, as far as the states of the band edges are not spuriously in the vicinity of the solute/defect energy levels (an instance which typically occurs at the GGA level).[32–34] Our piece-wise linear functional correctly describes the relative position of the VB of THF and of the solute occupied states (see main text). Decreasing the fraction of Fock exchange may result in a shrinking of the gap and the occurrence of aforementioned spurious interactions between solute and solvent states. A larger fraction of Fock exchange would bring to negligible differences in the *absolute* position of the energy levels but would deteriorate the accord with the relative position of the redox potentials and the THF band edges. Nevertheless, we here recalculate the vertical energy gap associated with vertical reduction of NAP in solution for a structural configuration, considering smaller and larger values ($\pm 5\%$) of α. Differences in the calculated vertical energy gap with respect to the piece-wise linear functional, which has been employed throughout this study, are below 0.02 eV, thus confirming the trends observed in the literature.[32,33]

Finally, we also evaluate the convergence of the calculated orbital energies with respect to the ADMM basis set size. Again, we consider the model of the neutral NAP solution, we recalculate the Kohn-Sham energy level associated with the HOMO for a structural

configuration, considering the larger FIT3 which is still affordable, even if at an increased computational cost (twice the computational time with the same number of cores, for the system under study). The difference in the calculated HOMO is as small as 0.03 eV, thus indicating that our choice of the cFIT3 basis is an excellent compromise between accuracy and computational time.

Electrostatic finite-size effects

In the main text, we report on the calculation of total-energy differences between charged and neutral supercells, which needs to be corrected for the electrostatic finite-size error affecting periodic DFT calculations. In particular, $E_{\text{corr}}(-, R_{-})$ has been defined in the main text as a correction to the energy of the charged NAP^{-} a charge in a periodic supercell, at the coordinates R_{-}, i.e. including both solute and solvent response. This correction ensues from the fact that the finite size of the supercell causes unrealistically large long-range electrostatic interactions between the NAP$-$ molecule in different periodic replicas. This in turn affects the calculated total energy and, hence, the electronic energy levels. Although a neutralizing background is usually adopted in periodic DFT calculations with extra charges,[35] this does not eliminate the interaction between the charge and its periodic replicas. Actually, both the interaction energy between the charge and its periodic replicas and the interaction of the charge with the background neutralizing charge in the reference cell must be corrected.

$E_{\text{corr}}(-, R_{-})$ term features two contributions: (i) the Madelung energy, accounting for the monopole charge subject to periodic boundary conditions and screened by the static dielectric constant ϵ_0 of the bulk medium (we here consider the experimental dielectric constant of ℓ-THF, $\epsilon_0(\text{THF} = 7.4$[36]); (ii) an alignment-like term due to the finite extent of the charge distribution.[37–39] $E_{\text{corr}}(-, R-)$ is here evaluated via the scheme first developed by Freysoldt, Neugebauer, and Van de Walle (FNV).[37–39] Similarly, the term $E_{\text{corr}}(-, R_0)$ corrects the energy of the vertically charged supercell after injection of an electron into neutral NAP (R_0). In this case, the charge is considered to be screened by the high-frequency dielectric

constant ϵ_∞ of THF. Again, we adopt the experimental $\epsilon_\infty(\text{THF}) = 1.75$, evaluated from the square of the refractive index.[40] Finally, $E_{\text{corr}}(0, R_-)$ corrects the total energy of neutral supercell achieved upon vertical removal of an electron. In fact, it has been demonstrated that, notwithstanding the formal charge neutrality, the ionic polarization charge, still present when the coordinates are fixed to those of the charged system, introduces an electrostatic finize-size error.[39,41] This is here corrected employing the method developed in Ref. 39.

We pinpoint that correction schemes employed in this work have been largely tested and employed on a variety of solid-state,[37–39,42] and more recently, liquid systems,[22,41] showing how the correction on moderately sized supercell provides corrected energies in excellent agreement with those achieved via linear extrapolation of the supercell energy at infinite size. Furthermore, we note that the use of experimental dielectric constants entails minimal differences with respect to ab initio estimated values, e.g. DFT-derived quantities in Ref. 43.

All calculated corrections are reported in Table S1.

Table S1: Electrostatic finite-size corrections for the periodic cubic supercell (a = 20 Å) of ℓ-THF including a solute molecule, employed in this work

Correction	Value
$E_{\text{corr}}(0, R_-)$	+0.09
$E_{\text{corr}}(0, R_-)$	+0.35
$E_{\text{corr}}(0, R_-)$	+0.26

Density Functional Theory Calculations with Implicit Solvent Models

DFT calculations for NAP and NAP^- with an implicit description of the solvent are carried out with the Gaussian16 software.[10] We employ the the B3LYP functional with the 6-31+G(d,p) basis set. Dielectric effects are included in the computations by using the polarizable continuum model (PCM).[44] Solvent reorganization energy is evaluated using the non-equilibrium PCM method, as implemented in Gaussian16.[10]

S2 Analysis for the radial distribution function of ℓ−THF

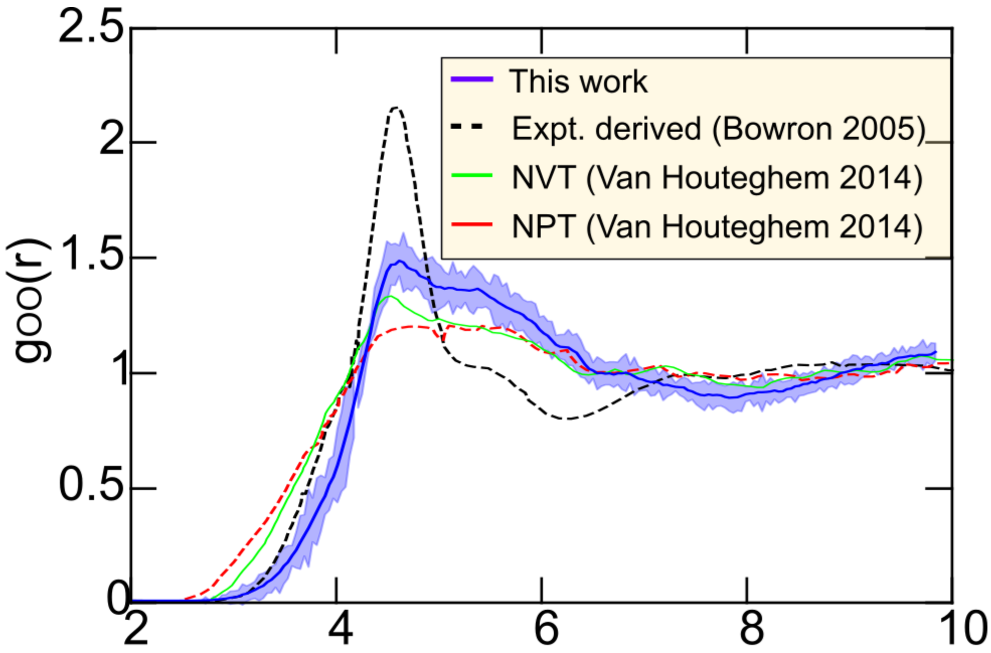

Figure S1: Oxygen-Oxygen radial distribution function for ℓ-THF from the present MD, compared with previous experimental and computational studies.[45,46] Shaded regions represent the standard deviation for each bin.

To evaluate whether the initial shift in the O–O RDF is statistically significant, if compared with previous computational work, we perform a block analysis of the trajectory, dividing it into 10 segments and computing the RDF for each block. The resulting RDFs are used to calculate the average and standard deviation σ at each distance bin. As shown in Figure S1 (with shaded $\pm\sigma$ regions), the initial rise of our RDF is slightly shifted compared to other computational results. As mentioned in the main text, we attribute the difference to slight variations in simulation protocols and to the fact that we use classical force-fields, even though fitted against QM calculations.

S3 Convergence of the blocking analysis and statistical error

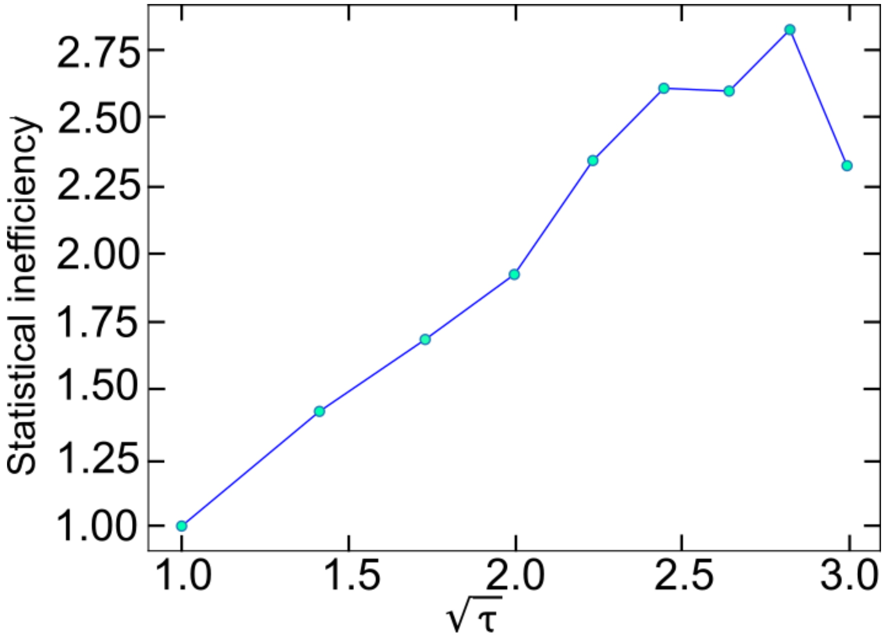

Figure S2: Statistical inefficiency plotted as a function of the square root of block size, $\sqrt{\tau}$, as obtained from blocking analysis of 100 energy values. Statistical inefficiency is calculated as $\mathrm{s} = \frac{\tau\sigma_\tau^2}{\sigma^2}$, where σ_τ^2 represents the variance of the block and σ^2 the sample variance. The curve increases due to autocorrelations at small block sizes, and ideally approaches a plateau region as blocks become statistically independent. A suitable value for the statistical inefficiency is selected by choosing either the plateau value or the last (i. e. the highest) available value from the plot, thus allowing for the calculation of the statistical error.

Blocking analysis is conducted on the calculated values in order to quantify the statistical error associated with potentially autocorrelated data. The time series is subdivided into non-overlapping blocks of increasing size τ, and for each of them the variance of the block mean is calculated. This variance, normalized by the sample variance, σ^2, yields an estimate of the statistical inefficiency, s, which in turn reflects the degree of correlation among data as a function of the block size τ. A plot of s vs $\sqrt{\tau}$ is here shown, for example, for the calculated vertical energy gap associated with NAP reduction, see Figure S2. This allows identification of a suitable value for s in the eventual plateau region (i. e. the convergence of the statistical inefficiency parameter). Finally, the statistical error of the sample mean is

then calculated as $\sqrt{\frac{s\sigma^2}{N}}$. As mentioned in the main text, with this procedure, we estimate statistical errors below 0.05 eV. In the case of the example, we achieve a statistical error of 0.02 eV. We also report the average energy differences inferred from different partitions of the total simulation time, showing that these are consistent with the global average and its statistical error, Figure S3.

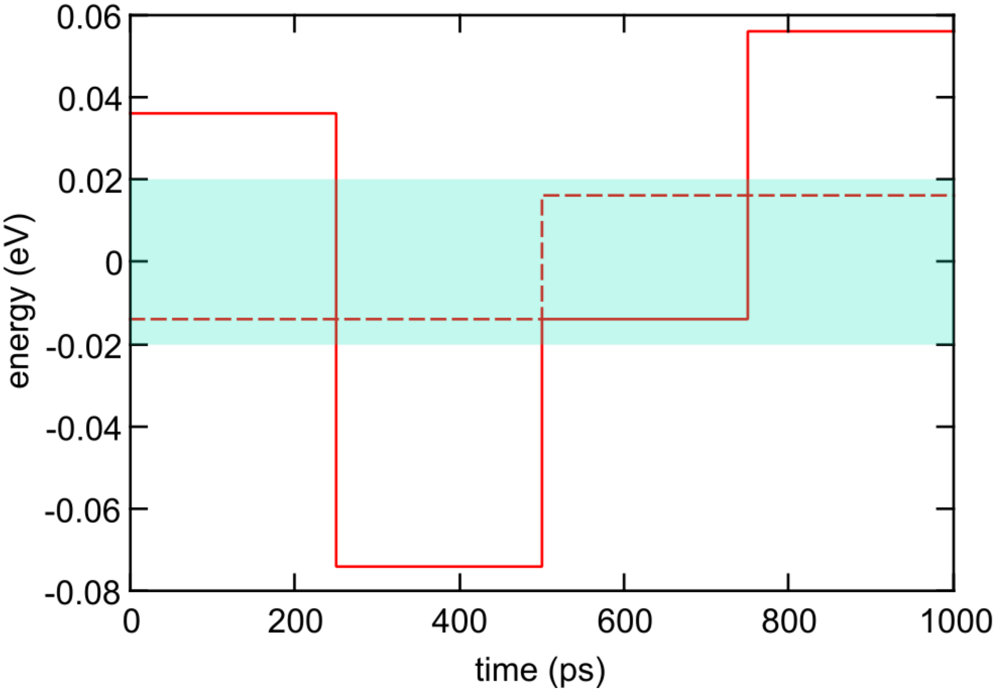

Figure S3: Average vertical energy differences evaluated inferred from different partitions of the total simulation time. The zero of energy has been set in correspondence of average over the complete simulation and the shaded cyan region represent the associated statistical error. Red solid and dashed lines are the average energies computed from quarters and halves of the total simulation, respectively.

S4 Tests on THF conformers and on THF dimers

The THF molecule is a peculiar case as it has two conformers, energetically separated by an exiguous difference. The so-called twisted conformer with C_s symmetry is found to be more stable of the envelope structure (cf. Figure S4) displaying C_2 symmetry by only a few meV, by both advanced *ab initio* calculations[47] and measurements.[48,49] We here calculate the energy difference between the two conformers, performing electronic-structure calculations of the isolated molecules at the KC+rVV10 level of theory, and, for comparison at (i) the KC, (ii) at the standard PBE0 level, and with the semi-local (iii) PBE and (iv) rVV10 functionals. The results are collected in Table S2, where we also include the energy differences calculated with the larger triple-ζ (cc-TZ) basis set.[50]

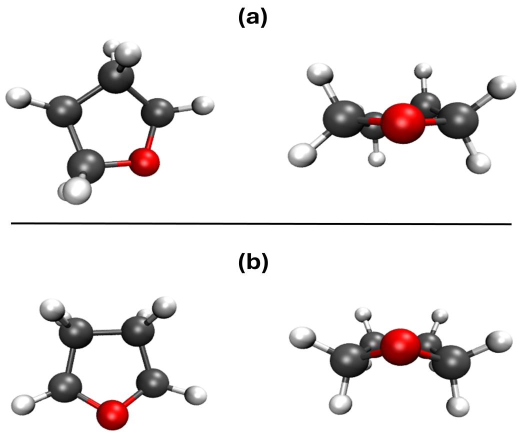

Figure S4: Stick&ball representation of the C_2 (a) and C_S (b) conformers of tetrahydrofuran.

Table S2: $\Delta E = E(C_2) - E(C_s)$, energy difference between C_2 and C_s conformers of THF molecule. All energies are in eV. Structures are shown in Figure S4. Reference values at the MP2 level of theory range between 0.007 eV and 0.01 eV, depending on the basis set.[47]

Basis-set	Functional				
	KC	KC+rVV10	PBE0	PBE	rVV10
MOLOPT	0.0180	0.0180	0.007	-0.007	-0.005
cc-TZ	0.0224	0.0225	0.0116	-0.0006	0.001

The results show that the hybrid functional is necessary to retrieve the expected energetics, as semi-local methods fail in correctly ordering the two conformers. The best results

are obtained with the hybrid KC scheme, as we estimate energy differences of ≈0.02 eV, in line with previous results. We note that employing a larger basis set has a small effect on the calculated values, while the inclusion of vdW interactions is not relevant in this case.

At variance with this, vdW interactions are likely to play a significant role in intermolecular interactions, which define the structural and the electronic properties of ℓ-THF. For this reason, we test the performance of the constructed functional in evaluating the energy differences between different structural configuration of THF dimers reported in Ref. 51, cf. Figure S5. In this case, we limit our comparative analysis to the hybrid KC functional with and without vdW interactions, again implemented via the rVV10 scheme, and we employ the MOLOPT basis set. From the relative energy differences of the THF dimers presented in Table S3, it is clear that vdW are fundamental to distinguish the different structures. In fact, the pure hybrid functional is not capable of differentiating the intermolecular interactions and, as a consequence, all the configurations appear to be almost iso-energetic, with an energy difference as small as 0.021 eV between the most and least stable structures. In contrast, when vdW interactions are included, the energy range is almost fivefold increased (0.1 eV). In particular, molecules lying on the same plane or stacked on top of each other with O atoms on opposite positions are found to be slightly more stable than other configurations in which the molecules are staggered or almost perpendicular.

Table S3: Total energies of the different THF dimers, referred to that of the a configuration, cf. Figure S5. All values are given in eV.

Dimer	KC	KC+rVV10	Ref. 51
a	0.0	0.0	0.0
b	0.000	0.079	0.003
c	0.002	0.080	0.004
d	0.004	0.082	0.006
e	0.005	0.084	0.014
f	0.006	0.087	0.056
g	0.013	0.093	0.071
h	0.021	0.100	0.082

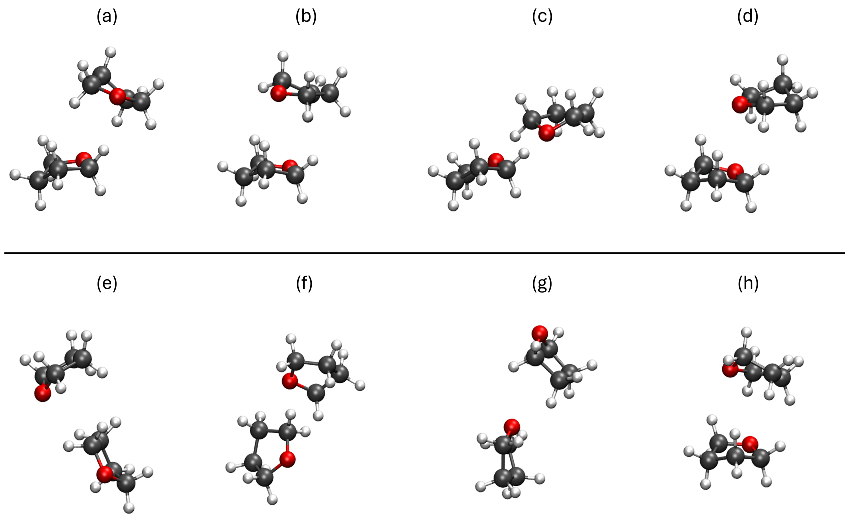

Figure S5: Stick&ball representation of different configurations for THF dimers, as reported in Ref. 51.

S5 Lowest occupied molecular orbital of NAP in ℓ-THF

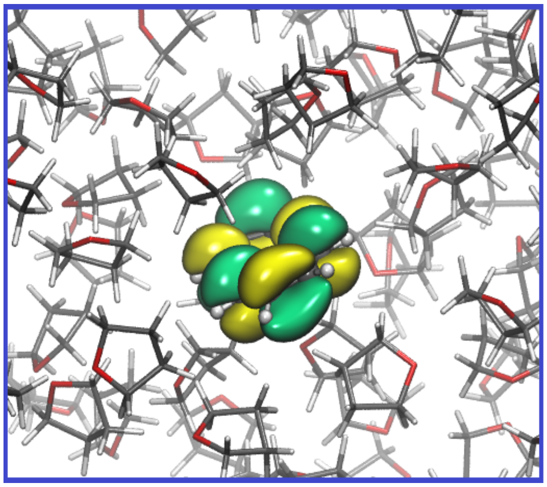

Figure S6: Isodensity representation (isovalue 0.01) of the lowest unoccupied molecular orbital of NAP in ℓ-THF. C atoms in gray, O in red and H in white.

S6 Spin density of a representative configuration for NAP^- in ℓ-THF

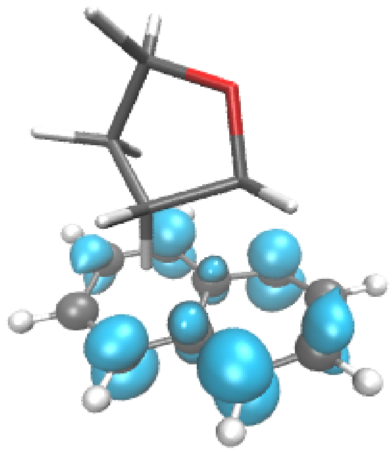

Figure S7: Isodensity representation (isovalue 0.005) of the spin density of NAP^- in ℓ-THF. C atoms in gray, O in red and H in white. The closest THF molecule is also shown.

S7 Dimer model for NAP-THF interactions

To approximately quantify the contribution to the solvent reorganization energy of the THF molecule getting closer to the solute [cf. Figure 3 (b) of the main text], we extract a THF-NAP^- dimer from the MD of NAP^-, considering the closest solvent molecule, representative of the shoulder in the RDF. We then perform a series of structural relaxations in which we constrain the CM-CM distance between the NAP^- and the THF molecule, see Figure S8. In particular, we scan the range of distances between 3.5 and 6 Å and determine the distance-dependent energy profile, see Figure S8 (c). This features a minimum at 4.25 Å, close to the position of the small RDF peak, which is at 0.27 eV below the energy of the dimer separated by 6 Å, i.e. similar to the average CM-CM distance of the neutral solution. We then repeat the same procedure considering the neutral charge state for the same structural configurations. In this case, the energy profile is found to display purely vdW interactions

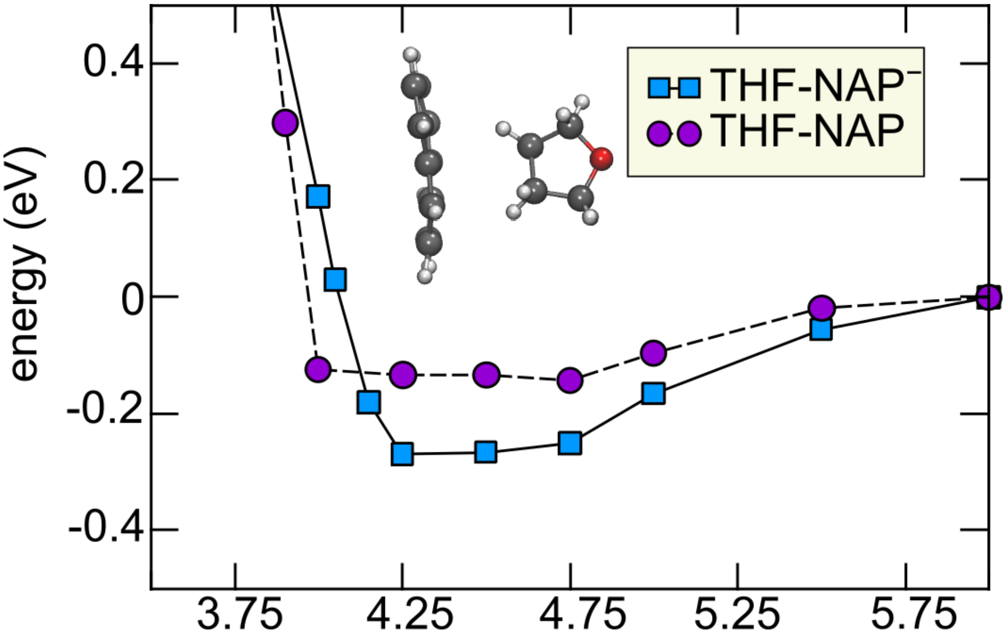

Figure S8: Energy profile for the THF-NAP and THF-NAP$^-$ dimers (cf. main text) as a function of the CM-CM distance. For each dimer, energies are referred to those calculated at a distance of 6 Å.

with a very shallow energy minimum shifted towards higher distances (4.75 eV). In this case, the energy difference between the two reference structures at 4.25 Å and 6 Å is only 0.13 eV. From this analysis, we roughly estimate that the fractions of $\lambda_{\text{ext}}(\text{ox})$ and $\lambda_{\text{ext}}(\text{red})$ deriving from single-molecule polarization as 0.27 eV and 0.13 eV, respectively, thus representing a sizable contribution to the overall reorganization energy.

S8 Definition of the angle between the solute and the THF dipole moment

We here report on the evaluation of the quantities defined in Figure 3 (c) of the main text. We first define the molecular plane: for each snapshot, the plane is evaluated using a regressive approach, specifically the linear least squares method. We center the coordinates on the

center of mass of the solute and we calculate the covariance matrix:

$$\Sigma = \frac{1}{N-1}\sum_i (r_i - r_{\mathrm{CM}})(r_i - r_{\mathrm{CM}})^T. \tag{S2}$$

We evaluate the eigenvectors and eigenvalues of this matrix, corresponding to the direction and variance of the data distributions, respectively. Thus, the best-fit plane is associated with the eigenvector corresponding to the minimum eigenvalue (i.e., the direction of minimum variance in the data distribution).

We then define θ as the angle between the normal vector to the plane, $\overrightarrow{n}$, and the THF molecular dipole $\overrightarrow{\mu}$, calculated using the partial charges adopted in classical MD simulations. This is given by the following equation:

$$\theta = \cos^{-1}\left(\frac{\overrightarrow{n}\cdot\overrightarrow{\mu}}{|n||\mu|}\right)\mathrm{sign}[\overrightarrow{n}\cdot\overrightarrow{r}_{\mathrm{CM}}(\mathrm{THF})], \tag{S3}$$

as illustrated in the main text. The ϕ angle is the complementary of θ. We discern between dipole moment pointing away or towards the molecular plane considering the sign of the scalar product between $\overrightarrow{n}$ and the vector pointing from the origin towards the center of mass of the considered THF molecule, $\overrightarrow{r}_{\mathrm{CM}}(\mathrm{THF})$.

S9 Distribution of the ϕ angle for the closest solvent molecules to solvated NAP^-

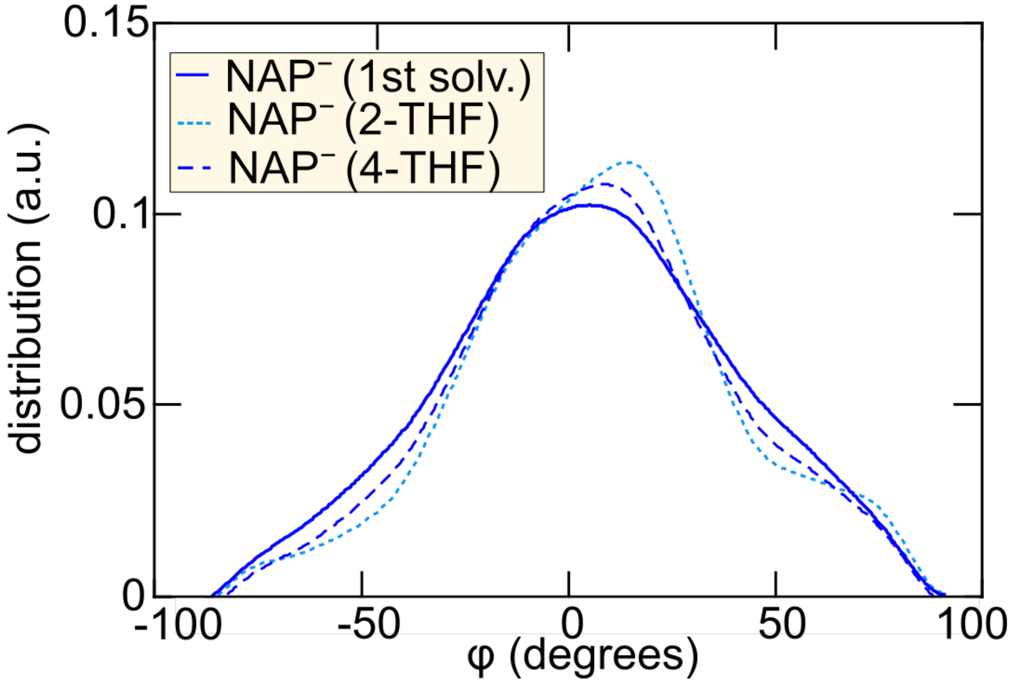

Figure S9: Distributions of the ϕ angle including only the 4 and the 2 THF molecules closest to the solute, in comparison with the distribution achieved for the full solvation shell.

S10 Time evolution of the solvent reorganization

In order to approximately evaluate the time required for the solvent to reorganize upon ET, we perform non-equilibrium MD simulations, in which we start from a representative structural configuration of the neutral NAP (anion NAP^-), vertically inject (detach) an electron and let the system evolve for 100 ps. Then, we monitor the time-dependent shift in the peak of the RDF between the O atoms of THF and the centers of mass of the NAP molecule. To this end, we evaluate the RDF for separated blocks of the MD simulation, until we observe convergence towards the equilibrium values. For the reduction process, cf. Figure S10 (a), we observe convergence of the distances, within a few ps of simulation, as the

molecules swiftly rearrange to point their O atoms away from the negatively charged anion. In contrast, the time-scale associated with reorganization upon ionization of NAP^- appear to be sensitively longer, cf. Figure S10 (b).

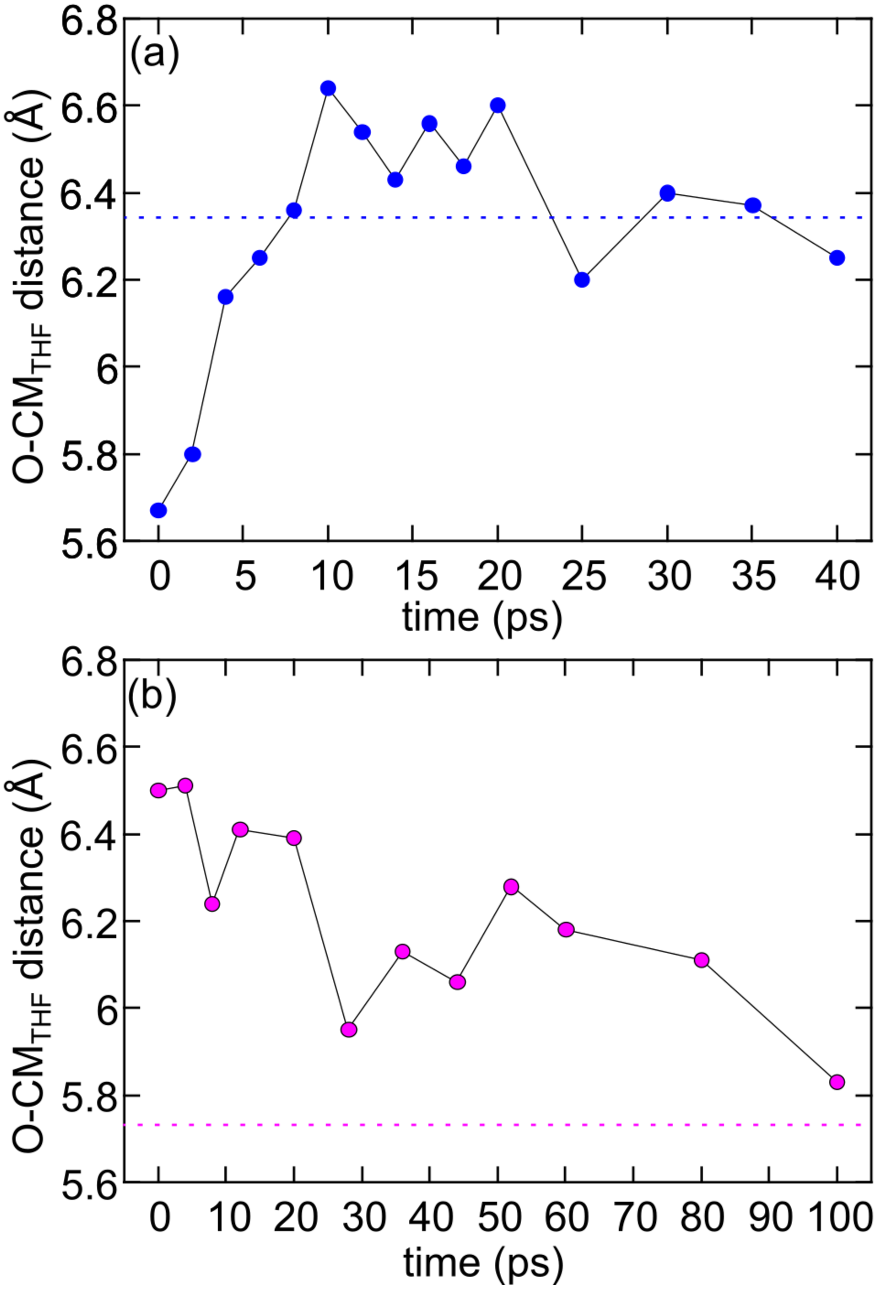

Figure S10: Time evolution of the position of the first peak of the RDF between the oxygen atoms of THF and the CM of the solute upon (a) reduction of NAP and (b) oxidation of NAP^-. Dashed lines indicate the equilibrium values.

References

(1) Landi, A.; Padula, D. Multiple Charge Separation Pathways in New-Generation Non-Fullerene Acceptors: a Computational Study. *J. Mater. Chem. A* **2021**, *9*, 24849–24856.

(2) Padula, D.; Landi, A.; Prampolini, G. Assessing alkyl side chain effects on electron transport properties of Y6-derived non-fullerene acceptors. *Energy Adv.* **2023**, *2*, 1215–1224.

(3) Landi, A.; Padula, D.; Peluso, A. Fast Nonradiative Decay Paths in Organic Solar Cells: Implications for Designing More Efficient Photovoltaic Systems. *ACS Appl. Energy Mater.* **2024**, *7*, 707–714.

(4) Ambrosio, F.; Wiktor, J.; Landi, A.; Peluso, A. Charge Localization in Acene Crystals from Ab Initio Electronic Structure. *J. Phys. Chem. Lett.* **2023**, *14*, 3343–3351.

(5) Marsalek, O.; Elles, C. G.; Pieniazek, P. A.; Pluhařová, E.; VandeVondele, J.; Bradforth, S. E.; Jungwirth, P. Chasing charge localization and chemical reactivity following photoionization in liquid water. *J. Chem. Phys.* **2011**, *135*.

(6) Ambrosio, F.; Pasquarello, A. Reactivity and Energy Level of a Localized Hole in Liquid Water. *Phys. Chem. Chem. Phys.* **2018**, *20*, 30281–30289.

(7) Giannini, S.; Martinez, P. M.; Semmeq, A.; Galvez, J. P.; Piras, A.; Landi, A.; Padula, D.; Vilhena, J.; Cerezo, J.; Prampolini, G. JOYCE3. 0: A General Protocol for the Specific Parametrization of Accurate Intramolecular Quantum Mechanically Derived Force Fields. *J. Chem. Theory Comput.* **2025**, *21*, 3156–3175.

(8) Cerezo, J.; Prampolini, G.; Cacelli, I. Developing Accurate Intramolecular Force Fields for Conjugated Systems Through Explicit Coupling Terms. *Theor. Chem. Acc.* **2018**, *137*, 80.

(9) Becke, A. D. Density-functional thermochemistry. III. The role of exact exchange. *J. Chem. Phys.* **1993**, *98*, 5648–5652.

(10) Frisch, M. J. et al. Gaussian 16 Revision C.01. 2016; Gaussian Inc. Wallingford CT.

(11) Jorgensen, W. L.; Maxwell, D. S.; Tirado-Rives, J. Development and Testing of the OPLS All-Atom Force Field on Conformational Energetics and Properties of Organic Liquids. *J. Am. Chem. Soc.* **1996**, *118*, 11225–11236.

(12) Bayly, C. I.; Cieplak, P.; Cornell, W.; Kollman, P. A. A well-behaved electrostatic potential based method using charge restraints for deriving atomic charges: the RESP model. *J. Phys. Chem.* **1993**, *97*, 10269–10280.

(13) Abraham, M. J.; Murtola, T.; Schulz, R.; Páll, S.; Smith, J. C.; Hess, B.; Lindahl, E. GROMACS: High Performance Molecular Simulations Through Multi-Level Parallelism from Laptops to Supercomputers. *SoftwareX* **2015**, *1-2*, 19–25.

(14) Darden, T.; York, D.; Pedersen, L. Particle mesh Ewald: AnN·log(N) Method for Ewald Sums in Large Systems. *J. Chem. Phys.* **1993**, *98*, 10089–10092.

(15) Hess, B.; Bekker, H.; Berendsen, H. J. C.; Fraaije, J. G. E. M. LINCS: A Linear Constraint Solver for Molecular Simulations. *J. Comput. Chem.* **1997**, *18*, 1463–1472.

(16) Berendsen, H. J. C.; Postma, J. P. M.; van Gunsteren, W. F.; DiNola, A.; Haak, J. R. Molecular dynamics with coupling to an external bath. *J. Chem. Phys.* **1984**, *81*, 3684–3690.

(17) Adamo, C.; Barone, V. Toward Reliable Density Functional Methods without Adjustable Parameters: The PBE0 Model. *J. Chem. Phys.* **1999**, *110*, 6158–6170.

(18) Perdew, J. P.; Ernzerhof, M.; Burke, K. Rationale for Mixing Exact Exchange with Density Functional Approximations. *J. Chem. Phys.* **1996**, *105*, 9982–9985.

(19) Bischoff, T.; Reshetnyak, I.; Pasquarello, A. Adjustable Potential Probes for Band-Gap Predictions of Extended Systems through Nonempirical Hybrid Functionals. *Phys. Rev. B* **2019**, *99*, 201114.

(20) Bischoff, T.; Wiktor, J.; Chen, W.; Pasquarello, A. Nonempirical Hybrid Functionals for Band Gaps of Inorganic Metal-Halide Perovskites. *Phys. Rev. Mater.* **2019**, *3*, 123802.

(21) Bischoff, T.; Reshetnyak, I.; Pasquarello, A. Band gaps of Liquid Water and Hexagonal Ice through Advanced Electronic-Structure Calculations. *Phys. Rev. Research* **2021**, *3*, 023182.

(22) Ambrosio, F.; Capobianco, A.; Landi, A.; Pizza, T.; Peluso, A. Is a thin mechanism appropriate for aromatic nitration? *Phys. Chem. Chem. Phys.* **2023**, *25*, 2359–2365.

(23) Vydrov, O. A.; Van Voorhis, T. Nonlocal van der Waals Density Functional: The Simpler the Better. *J. Chem. Phys.* **2010**, *133*, 244103.

(24) Sabatini, R.; Gorni, T.; de Gironcoli, S. Nonlocal van der Waals Density Functional Made Simple and Efficient. *Phys. Rev. B* **2013**, *87*, 041108.

(25) Ambrosio, F.; Miceli, G.; Pasquarello, A. Structural, dynamical, and electronic properties of liquid water: A hybrid functional study. *The Journal of Physical Chemistry B* **2016**, *120*, 7456–7470.

(26) Guidon, M.; Hutter, J.; VandeVondele, J. Robust Periodic Hartree-Fock Exchange for Large-Scale Simulations Using Gaussian Basis Sets. *J. Chem. Theory Comput.* **2009**, *5*, 3010–3021.

(27) VandeVondele, J.; Krack, M.; Mohamed, F.; Parrinello, M.; Chassaing, T.; Hutter, J. Quickstep: Fast and Accurate Density Functional Calculations Using a Mixed Gaussian and Plane Waves Approach. *Comput. Phys. Commun.* **2005**, *167*, 103 – 128.

(28) Hartwigsen, C.; Goedecker, S.; Hutter, J. Relativistic Separable Dual-Space Gaussian Pseudopotentials from H to Rn. *Phys. Rev. B* **1998**, *58*, 3641–3662.

(29) VandeVondele, J.; Hutter, J. Gaussian Basis Sets for Accurate Calculations on Molecular Systems in Gas and Condensed Phases. *J. Chem. Phys.* **2007**, *127*, 114105.

(30) Guidon, M.; Schiffmann, F.; Hutter, J.; VandeVondele, J. Ab Initio Molecular Dynamics Using Hybrid Density Functionals. *J. Chem. Phys.* **2008**, *128*, 214104.

(31) Guidon, M.; Hutter, J.; VandeVondele, J. Auxiliary Density Matrix Methods for Hartree-Fock Exchange Calculations. *J. Chem. Theory Comput.* **2010**, *6*, 2348–2364.

(32) Alkauskas, A.; Broqvist, P.; Pasquarello, A. Defect Energy Levels in Density Functional Calculations: Alignment and Band Gap Problem. *Phys. Rev. Lett.* **2008**, *101*, 046405.

(33) Alkauskas, A.; Broqvist, P.; Pasquarello, A. Defect Levels through Hybrid Density Functionals: Insights and Applications. *Phys. Status Solidi B* **2011**, *248*, 775–789.

(34) Ambrosio, F.; Miceli, G.; Pasquarello, A. Redox Levels in Aqueous Solution: Effect of van der Waals Interactions and Hybrid Functionals. *J. Chem. Phys.* **2015**, *143*, 244508.

(35) Van de Walle, C. G.; Neugebauer, J. First-principles calculations for defects and impurities: Applications to III-nitrides. *Journal of applied physics* **2004**, *95*, 3851–3879.

(36) Metz, D. J.; Glines, A. Density, viscosity, and dielectric constant of tetrahydrofuran between-78 and 30. degree. *The Journal of Physical Chemistry* **1967**, *71*, 1158–1158.

(37) Freysoldt, C.; Neugebauer, J.; Van de Walle, C. G. Fully *Ab Initio* Finite-Size Corrections for Charged-Defect Supercell Calculations. *Phys. Rev. Lett.* **2009**, *102*, 016402.

(38) Komsa, H.-P.; Rantala, T. T.; Pasquarello, A. Finite-Size Supercell Correction Schemes for Charged Defect Calculations. *Phys. Rev. B* **2012**, *86*, 045112.

(39) Falletta, S.; Wiktor, J.; Pasquarello, A. Finite-size corrections of defect energy levels involving ionic polarization. *Phys. Rev. B* **2020**, *102*, 041115.

(40) Critchfield, F. E.; Gibson Jr, J. A.; Hall, J. L. Dielectric constant and refractive index from 20 to 35° and density at 25° for the system tetrahydrofuran—Water1. *Journal of the American Chemical Society* **1953**, *75*, 6044–6045.

(41) Ambrosio, F.; Miceli, G.; Pasquarello, A. Electronic levels of excess electrons in liquid water. *The journal of physical chemistry letters* **2017**, *8*, 2055–2059.

(42) Komsa, H.-P.; Broqvist, P.; Pasquarello, A. Alignment of Defect Levels and Band Edges through Hybrid Functionals: Effect of Screening in the Exchange Term. *Phys. Rev. B* **2010**, *81*, 205118.

(43) Capobianco, A.; Wiktor, J.; Landi, A.; Ambrosio, F.; Peluso, A. Electron Localization and Mobility in Monolayer Fullerene Networks. *Nano Letters* **2024**, *24*, 8335–8342.

(44) Tomasi, J.; Mennucci, B.; Cammi, R. Quantum mechanical continuum solvation models. *Chemical reviews* **2005**, *105*, 2999–3094.

(45) Bowron, D. T.; Finney, J. L.; Soper, A. K. The structure of liquid tetrahydrofuran. *J. Am. Chem. Soc.* **2006**, *128*, 5119–5126.

(46) Van Houteghem, M.; Ghysels, A.; Verstraelen, T.; Poelmans, W.; Waroquier, M.; Van Speybroeck, V. Critical Analysis of the Accuracy of Models Predicting or Extracting Liquid Structure Information. *J. Phys. Chem. B* **2014**, *118*, 2451–2470.

(47) Rayón, V. M.; Sordo, J. A. Pseudorotation motion in tetrahydrofuran: An ab initio study. *J. Chem. Phys.* **2005**, *122*.

(48) Geise, H.; Adams, W.; Bartell, L. S. Electron diffraction study of gaseous tetrahydrofuran. *Tetrahedron* **1969**, *25*, 3045–3052.

(49) Park, S. M.; Lee, Y. R.; Kim, H. L.; Kwon, C. H. Conformational structures of the tetrahydrofuran cation determined using one-photon mass-analyzed threshold ionization spectroscopy. *Phys. Chem. Chem. Phys.* **2017**, *19*, 30362–30369.

(50) Dunning, T. H. Gaussian Basis Sets for Use in Correlated Molecular Calculations. I. The Atoms Boron through Neon and Hydrogen. *J. Chem. Phys.* **1989**, *90*, 1007–1023.

(51) Majerz, I. Weak interactions in furan dimers. *J. Comput. Aided Mol. Des.* **2018**, *32*, 1247–1258.
